# Green Synthesis of Zinc Oxide Nanoparticles Using Chamomile and Green Tea Extracts and Evaluation of Their Anti-inflammatory and Antioxidant Activity: An In Vitro Study

**DOI:** 10.7759/cureus.46088

**Published:** 2023-09-27

**Authors:** Shubhangini Chatterjee, Jaiganesh R, Rajeshkumar S

**Affiliations:** 1 Department of Periodontics, Saveetha Dental College and Hospital, Saveetha Institute of Medical and Technical Sciences, Saveetha University, Chennai, IND; 2 Department of Pharmacology, Saveetha Dental College and Hospital, Saveetha Institute of Medical and Technical Sciences, Saveetha University, Chennai, IND

**Keywords:** antioxidant, anti-inflammatory, zinc oxide nanoparticles, green tea, chamomile tea

## Abstract

Background

An important field of study in contemporary material science is the synthesis of metallic nanoparticles. The wide range of uses for zinc nanoparticles in industries like diagnosis and antimicrobial catalysis has sparked particular interest in them. Plant extracts are used to synthesize zinc nanoparticles, opening up a wide range of potential applications. Hence, the current study aims to demonstrate the anti-inflammatory and antioxidant activity of zinc oxide nanoparticles mediated by green tea and chamomile tea combination.

Materials and methods

Leaves of green tea and chamomile tea were combined in a ratio of 1 gram each. To make a 1-molar solution of the extract, the weighted extracts were thoroughly mixed with 100 ml of distilled water in conical flasks. To synthesize nanoparticles, a magnetic stirrer and an orbital shaker were used alternately with an extract of chamomile and green tea, 0.016 g of zinc oxide, and 90 ml of distilled water at 900 rpm. By using an albumin denaturation assay, the synthesized nanoparticles' anti-inflammatory activity was assessed. Bovine serum albumin (BSA) and egg albumin (EA) were the reagents used in the assay. The antioxidant activity of zinc oxide nanoparticles, which is mediated by chamomile and green tea, was determined using 2,2-diphenyl-1-picrylhydrazyl (DPPH) and H_2_O_2 _(hydrogen peroxide) radical scavenging assays. An independent sample t-test was done to compare the anti-inflammatory and antioxidant potentials of zinc oxide nanoparticles mediated by green tea and chamomile tea combination and control using SPSS Statistics version 22.0 software (IBM Corp. Released 2013. IBM SPSS Statistics for Windows, Version 22.0. Armonk, NY: IBM Corp.), and any p-value less than 0.05 was considered statistically significant.

Results

In this study, the anti-inflammatory activity and antioxidant activity were assessed at variable concentrations of the reaction mixture. The combination of chamomile and green tea extracts mediated by zinc oxide nanoparticles at 50μl concentration showed the maximum anti-inflammatory activity and antioxidant activity at 87% inhibition, respectively.

Conclusion

Both assays successfully demonstrated better anti-inflammatory and antioxidant activity of zinc oxide nanoparticles mediated by chamomile and green tea combination when compared to control and, therefore, could be evaluated as a potential therapeutic agent.

## Introduction

Nanotechnology is a transformative technological advancement with the potential to revolutionize the field of science. Technology that allows for the molecular manipulation or self-assembly of such materials offers potentially game-changing opportunities in science, technology, and business [1]. One of the main subfields of nanotechnology, which supports the development of numerous new inventions, is the nanoparticle. A three-dimensional structure with a nanoscale measurement between one and one hundred nanometers (nm) is referred to as a nanoparticle [2]. Nanotechnology describes the pattern of how atoms come together and separate to form objects at various length scales [3]. Sustained interest in this field of study, especially in the realm of nanomedicine, is driven by the prospect of repurposing existing products to create novel, enhanced versions possessing improved features and characteristics. Because there are so many potential ways that nanotechnology could improve human life, there are high expectations for its use. Hence, it appears that the trend in biomedicine is moving toward the miniaturization of biomedical products. Therefore, metal-based nanomaterials stand out as a notable illustration of nanomaterials that have recently become an area of interest and have been applied widely. Since many organisms create metal-based nanoparticles when they detoxify heavy metals, this technology is not new, and in recent decades, it has been widely used in a variety of fields [4]. Sol-gel, chemical reduction, hydrothermal, laser ablation, ion sputtering, and many other physical and chemical processes can be used to produce metal nanoparticles [5]. These methods do, however, have a number of drawbacks, including the high cost of the instruments used, the need for skilled labor, and the fact that they are toxic to the environment.

Therefore, the green synthesis method, which uses plants and microorganisms, has emerged as the best technique for producing nanoparticles [6]. Green synthesis for creating nanoparticles is considered safer and more environmentally friendly because of its potential for stabilizing and reducing any potential damage to the environment.

Zinc oxide has drawn a lot of interest as a cost-effective, safe, and biocompatible material. Zinc oxide is recognized as the safest metal oxide by the US FDA [7]. Zinc is most recognized for its role in preserving interactions between proteins and nucleic acids within tissues and cells. When compared to other physiologic metals such as iron, cobalt, and manganese, zinc oxide exhibits significantly greater chemical stability [8]. Among the many engineering uses for zinc oxide nanoparticles are ultraviolet (UV) protection, pigments, chemical sensors, bio-molecular detection, diagnostics, and piezoelectric devices. Inflammatory-relieving, antibacterial, anti-cancer, and anti-diabetic properties of zinc oxide nanoparticles have also been demonstrated.

Since ancient times, the relationship between plants and human health has been studied [9]. For at least 5,000 years, herbs have held a pivotal role in both traditional and alternative forms of medical treatment [10]. Herbs typically have few toxic side effects, which may explain why they have remained popular for so long. The dried flowers of the *Matricaria* species are used to create standardized tea and one of the most popular medicinal herbs, chamomile. Chamomile has been suggested for use in a number of therapeutic procedures and is one of the oldest, most well-known, and best-documented medicinal plants in the world [11].

In various regions of the world, green or black tea made from the Camellia sinensis plant is drunk. However, out of all of these, green tea consumption has been linked to the healthiest effects on people [12]. Green tea may slow the spread of periodontal disease, according to some research. A large number of in vitro studies have concluded that the green tea catechin epigallocatechin gallate prevents the growth of *Porphyromonas gingivalis* and *Prevotella* species on human buccal epithelial cells [13]. It is also believed that green tea protects against inflammatory conditions by reducing the production of pro-inflammatory cytokines, particularly interleukin-8 (IL-8). This is possible due to its potent antioxidant and anti-inflammatory properties. It has also been found that the ability of gingival crevicular fluid to absorb antioxidants increases in response to green tea products.

Due to the many benefits of both chamomile and green tea, assessment of its anti-inflammatory and antioxidant properties can prove useful for the treatment of various oral lesions and in periodontal therapy. Hence, the current study aims to synthesize and then demonstrate the anti-inflammatory and antioxidant activity of zinc oxide nanoparticles mediated by green tea and chamomile tea combination.

## Materials and methods

Green synthesis of zinc oxide nanoparticles

**Figure 1:**
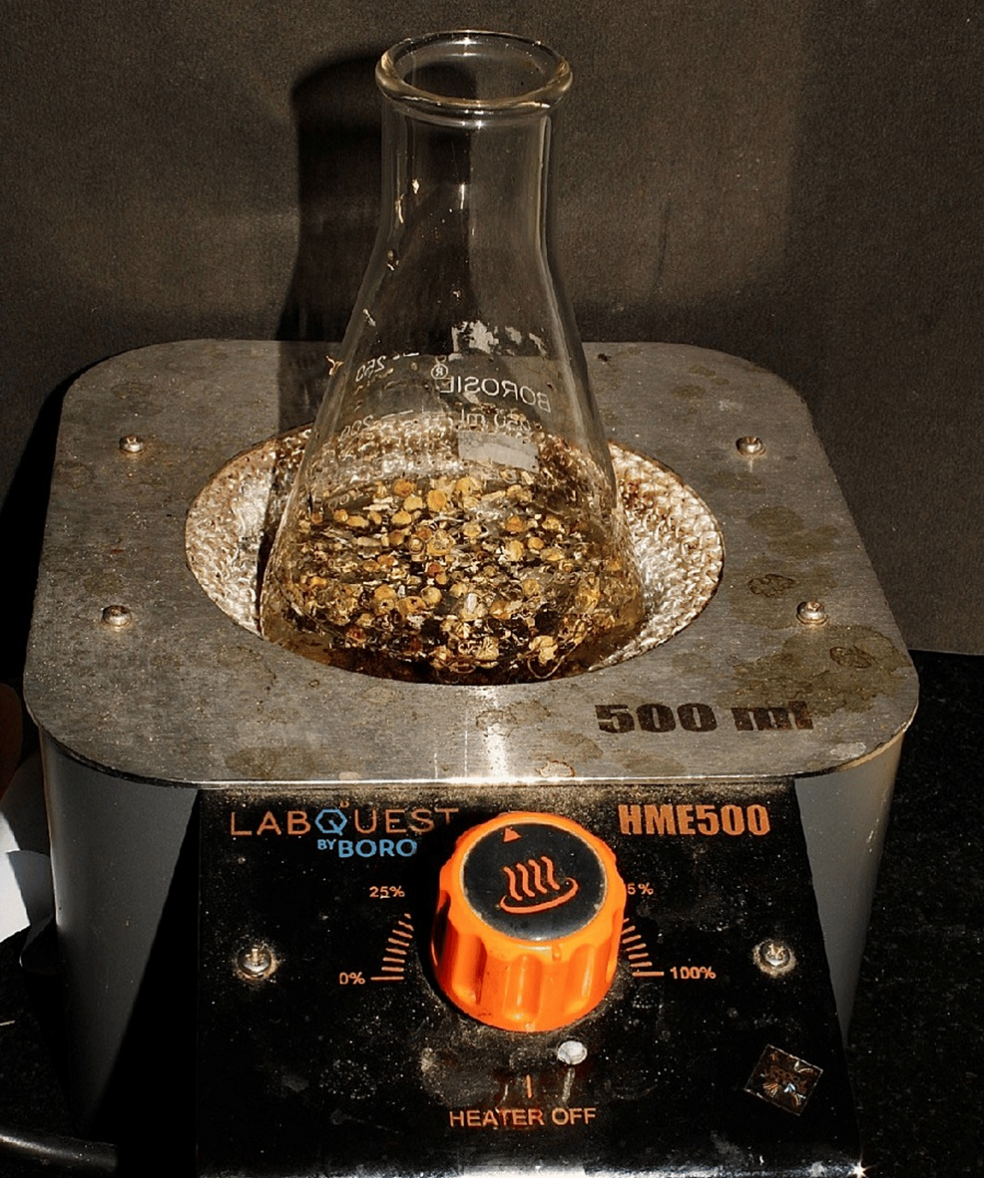
Mixture of green tea leaves and chamomile tea leaves Combination of commercially available green tea leaves and chamomile tea leaves

Purchased tea leaves, chamomile (Twinings) and green (Tetley), were thoroughly cleaned in distilled water. The leaves were then pounded into fine particles with a mortar and pestle and weighed separately. Green tea leaves were combined with chamomile tea leaves to make 1 gram of total tea leaves. Conical flasks containing weighted extracts were filled with distilled water (100 ml), and this was later used to dissolve the extracts (Figure 1).

**Figure 2:**
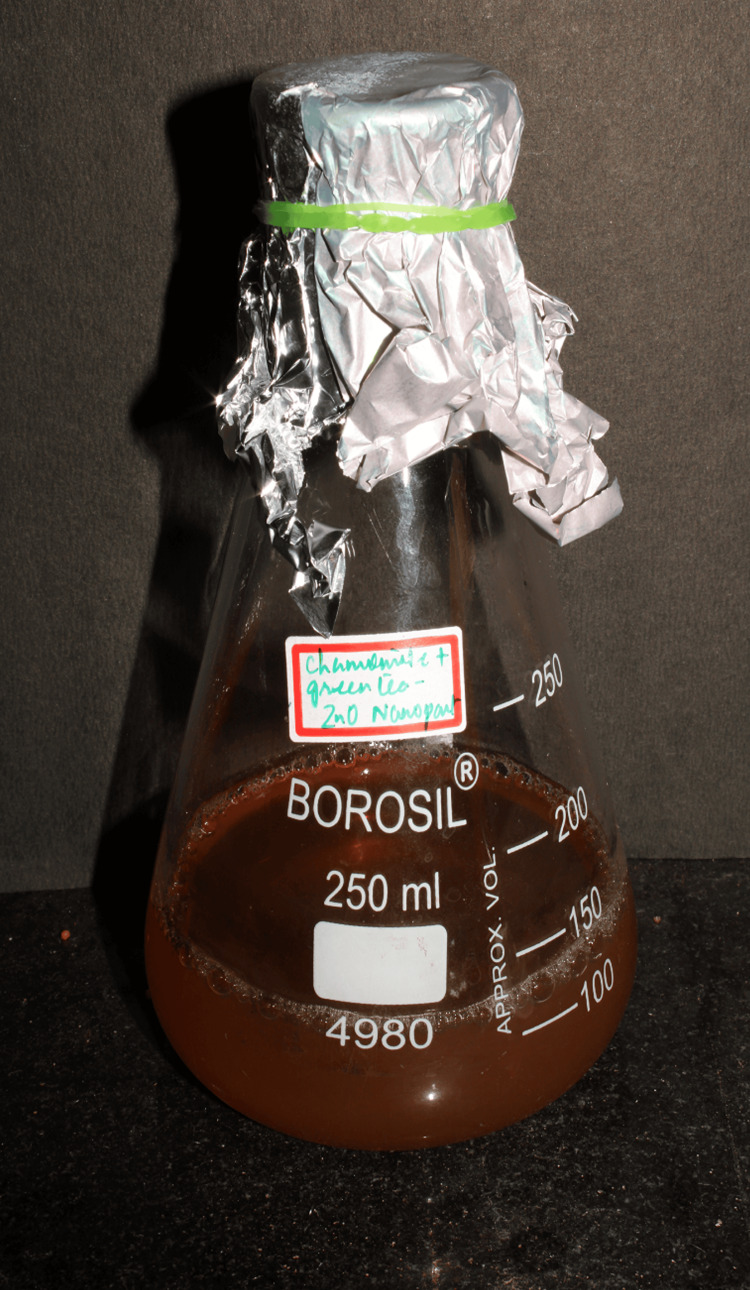
Formulation Prepared solution of zinc oxide nanoparticles formed using chamomile tea and green tea leaves

One molar solution of the extract was created by thoroughly mixing the mixture, and it was heated at 60 degrees Celsius for 15 to 20 minutes and filtered. The filtered chamomile and green tea extracts were mixed with 0.016 g of zinc oxide and 90 ml of distilled water and then alternately stirred and shaken at 900 rpm using a magnetic stirrer and an orbital shaker. Periodically, an observation was made to assess the color change of the solution, and it was photographed and recorded (Figure 2).

Evaluation of anti-inflammatory effects

Bovine Serum Albumin (BSA) Denaturation Assay

The anti-inflammatory effects of zinc oxide nanoparticles mediated by chamomile and green tea combination were evaluated using the inhibition of albumin denaturation assay. BSA was used. In animal serum, it accounts for nearly 60% of all the protein. BSA undergoes denaturation during heating, which causes it to begin producing an antigen linked to a type 3 hypersensitivity reaction that causes an inflammatory response. Then, 400 μl of chamomile and green tea-mediated zinc oxide nanoparticles were combined with 2 mL of bovine albumin fraction. It was distributed to different concentrations in a range of 10μl to 50μl. After mixing with 1N hydrochloric acid into the mixture, the solution's pH was changed to 6.8. Incubation of the reaction mixture was done for 20 minutes at room temperature, followed by 20 minutes in a water bath at 55 degrees Celsius. The components were then left unaltered to reach room temperature following the incubation process. It was evaluated for protein denaturation. The absorbance of the mixture was analyzed using a UV-spectrophotometer, which was set at 660 nm. The evaluation of anti-inflammatory activity was done using diclofenac sodium as a standard control, and it has known anti-inflammatory activity.

Calculation of the protein denaturation percentage:

% Inhibition = {Absorbance of control - Absorbance of sample} x 100 / Absorbance of control 

Egg Albumin (EA) Denaturation Assay

A 0.2 ml concentration of EA and 2.8 ml of phosphate-buffered saline were mixed, and a 5 ml solution was prepared. For the chamomile and green tea combination-mediated zinc oxide nanoparticles, specific concentrations were prepared separately. Diclofenac was used as a control. At a temperature of 37 degrees for 15 minutes, all prepared solutions were heated and then allowed to cool down to room temperature. This was done at an absorption of 660 nm, and measurements were then made.

Estimation of antioxidant activity

2,2-Diphenyl-1-Picrylhydarzyl (DPPH) Assay

A DPPH radical scavenging assay was used to assess the antioxidant potential of zinc oxide nanoparticles incorporated with chamomile and green tea. DPPH is a stable free radical, and it reacts with substances that can give off a hydrogen atom. It has a nitrogen center with a purple color and stable lipophilic free radicals. As a free radical, DPPH causes oxidation. In order to change the absorbance at 517 nm, the antioxidant can give an electron to the DPPH radical. Gradually, the color turned a light yellow. Each of the five test tubes received 2 ml of plant extract. Five test tubes were filled with 0.1 mm of DPPH solution and 50% of the methanol solution (buffer). In five test tubes, different concentrations of plant extract containing zinc oxide nanoparticles (10 μL to 50 μL) were added. Then, the mixture was kept in a dark area at room temperature for 30 minutes. At 517 nm, spectrophotometric analysis of the absorbance value was performed. The methanol solution served as the blank. As a control, a solution of methanol and 0.1 mM DPPH was used. The standard used was ascorbic acid. The IC50 value (minimum inhibitory concentration) was calculated. The calculation of the percentage of inhibition is as follows:

% inhibition = {Absorbance of control - Absorbance of sample} x 100 / Absorbance of control.

H_2_O_2_ (Hydrogen Peroxide) Assay

The iron-ethylenediaminetetraacetic acid (EDTA) solution, 0.5 mL of 0.018% EDTA, 1.0 mL of dimethyl sulfoxide (0.85% in 0.1 mol/L phosphate buffer pH 7.4), and 0.5 mL of 0.22% ascorbic acid made up the reaction mixture. The pre-made solutions were warmed in a water bath for 15 minutes at 80-90°C, and the reaction was halted by the addition of 1.00 mL of ice-cold trichloroacetic acid (17.5%). Three mL of Nash reagent containing 75.0 g of ammonium acetate, 3.0 ml of glacial acetic acid, 2.0 ml of acetylacetone, and 1 liter of distilled water were added to the mixture and incubated for 15 minutes at room temperature. A yellow color was noticed, and an assessment of intensity at 412 nm was done. Ascorbic acid was used as a standard. The amount of inhibition was determined.

Statistical analysis

The values were tabulated in Microsoft Excel (Microsoft Corporation, Redmond, WA) and transferred to SPSS Statistics version 22.0 (IBM Corp. Released 2013. IBM SPSS Statistics for Windows, Version 22.0. Armonk, NY: IBM Corp.) for statistical analysis. An independent t-test was carried out between the control and experimental groups at 10 μl, 20 μl, 30 μl, 40 μl, and 50 μl concentrations. Any p-value less than 0.05 was considered significant.

## Results

Estimation of anti-inflammatory activity

**Figure 3:**
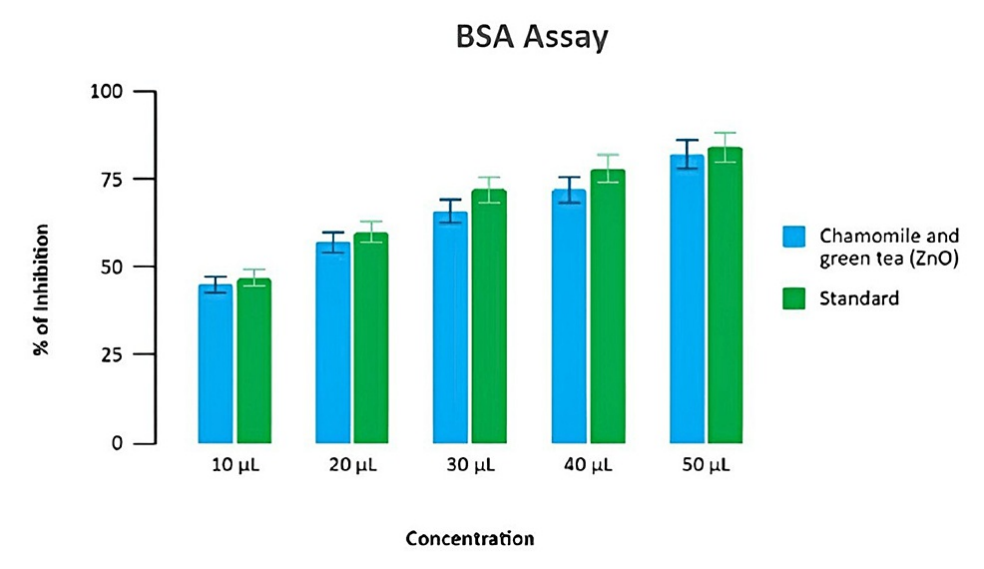
Anti-inflammatory potential of the prepared formulation (BSA assay) Percentage of inhibition test of chamomile-green tea combination-mediated zinc oxide nanoparticles and diclofenac sodium (standard) in BSA at different concentrations BSA: bovine serum albumin

Evaluation of anti-inflammatory activity was done at five different concentrations of the reaction mixture, which varied from 10 μL to 50 μL. Anti-inflammatory activity tests showed inhibition at 49%, 50%, 75%, 85%, and 87%, respectively. However, the standard solution showed a 90% inhibition. Our test extract mediated by zinc oxide nanoformulation at 50 μL of concentration exhibited the highest level of anti-inflammatory activity at 87% inhibition for BSA assay (Figure 3).

**Figure 4:**
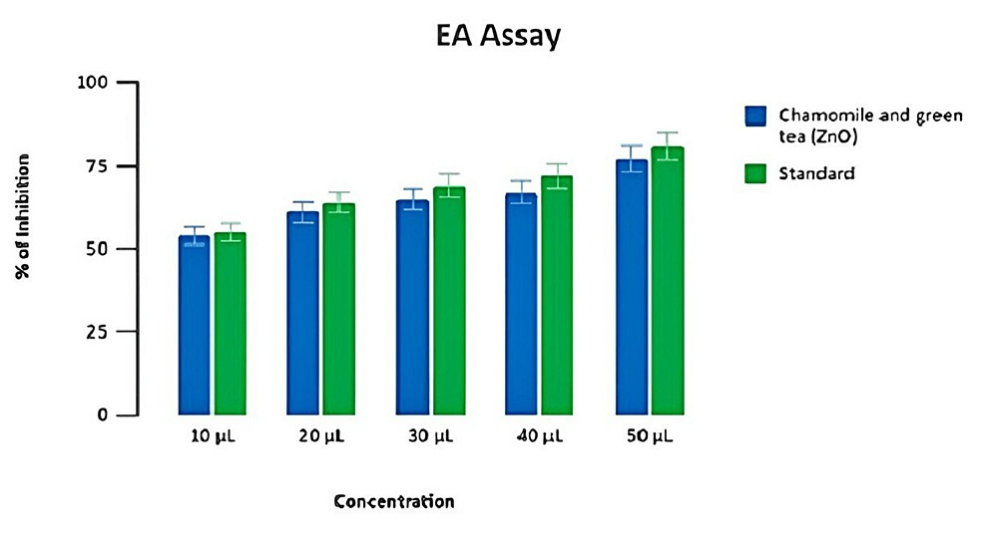
Anti-inflammatory potential of the prepared formulation (EA assay) Percentage of inhibition test of chamomile-green tea combination-mediated zinc oxide nanoparticles and diclofenac sodium (standard) in EA at different concentrations EA: egg albumin

Our test extract mediated by zinc oxide nanoformulation at 50 μL of concentration exhibited the highest level of anti-inflammatory activity at 87% inhibition for EA assay (Figure 4).

Estimation of antioxidant activity

**Figure 5:**
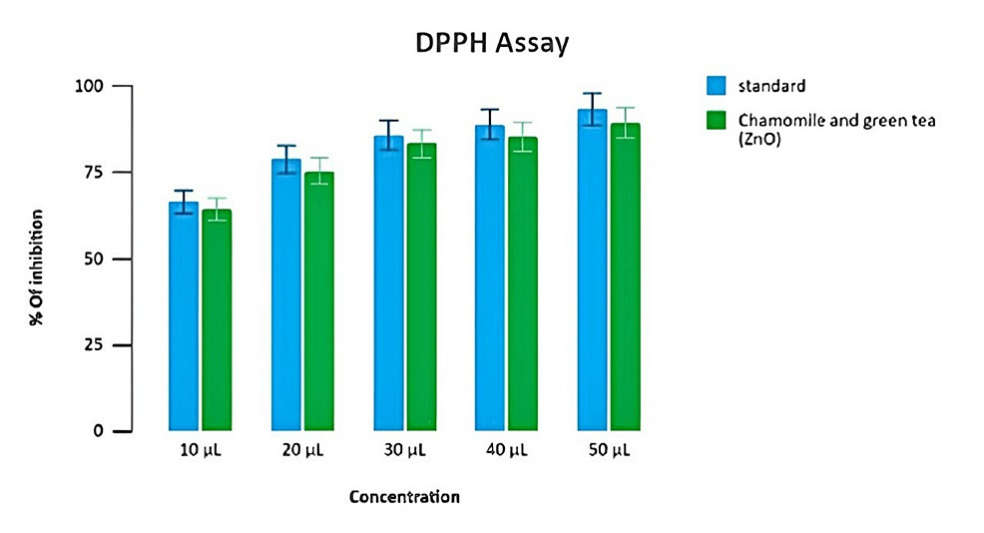
Antioxidant potential of the prepared formulation (DPPH assay) Percentage of inhibition test of chamomile-green tea combination-mediated zinc oxide nanoparticles in DPPH at different concentrations DPPH: 2,2-diphenyl-1-picrylhydarzyl

The antioxidant activity was evaluated for variable concentrations of reaction mixture ranging from 10μL to 50 μL. Antioxidant activity at different concentrations from 10 to 50 μL showed inhibition at 52%, 64%, 73%, 80%, and 87%, respectively. The standard solution showed 90% inhibition. Test extract mediated by zinc oxide nanoparticles at 50 μL of concentration exhibited the highest level of antioxidant activity at 87% inhibition, whereas it was noted at 90% inhibition for standard DPPH assay (Figure 5).

**Figure 6:**
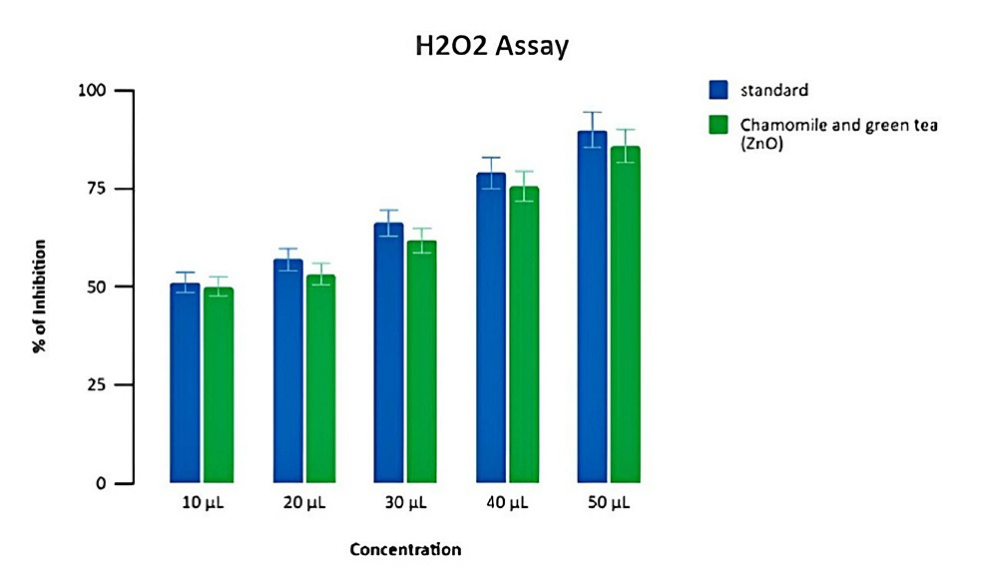
Antioxidant potential of prepared formulation (H₂O₂ assay) Percentage of inhibition test of chamomile-green tea combination-mediated zinc oxide nanoparticles in H₂O₂ assay at different concentrations H₂O₂: hydrogen peroxide

Test extract mediated by zinc oxide nanoparticles at 50 μL of concentration exhibited the highest level of antioxidant activity at 87% inhibition, whereas it was noted at 90% inhibition for standard H₂O₂ assay (Figure 6).

## Discussion

The present study aimed to evaluate the antioxidant and anti-inflammatory potentials of a herbal formulation of zinc oxide nanoparticle-mediated green tea and chamomile tea combination through in vitro assays. The findings of this study provide valuable insights into the therapeutic implications of the herbal formulation and its potential as a natural remedy for conditions associated with oxidative stress and inflammation. The anti-inflammatory and antioxidant properties of the formulation were assessed against the gold-standard diclofenac sodium and ascorbic acid as control, respectively, and it was found that the herbal formulation showed better properties at lower concentrations against the standard, and at higher values, its properties were comparable with the respective controls.

Many studies conducted on animal models have consistently demonstrated the multifaceted benefits of green tea, including its anti-inflammatory, antibacterial, antidiabetic, and notably, anticancer properties. Chamomile, which belongs to the Asteraceae family, owes its pharmaceutical properties to its flavonoid compounds and the breakdown of volatile oils. Extracts derived from this plant exhibit a wide range of pharmacological properties, encompassing anti-itching, anti-inflammatory, antimicrobial, antiviral, and antioxidant attributes [14].

Even though there are numerous ways to make nanoparticles, green synthesis is preferred over physical and chemical processes because it is straightforward, economical, and devoid of hazardous materials or toxic organic solvents. When nanoparticles are formed through physicochemical processes, expensive and dangerous chemicals are used. These processes also call for high temperatures and pressures and are hazardous to the environment. These nanoparticles cannot be used in biomedical settings because the toxic chemicals can occasionally remain adsorbed on the surface of the nanoparticle. Because of the expanding applications in the biomedical fields, producing nanoparticles using environmentally friendly methods has recently become a trending research topic. Zinc oxide nanoparticle synthesis has recently been reported using a number of green and biological methods [15]. Zinc oxide nanoparticles were created in this instance using green tea extract and chamomile tea extract. Using the catechins and flavonols in the tea extracts, zinc acetate is reduced to zinc oxide nanoparticles.

The approach for nanoparticle synthesis can be expanded for large-scale manufacturing, showcasing robust bioactivity, as exemplified by the production of metallic nanoparticles. These synthesized nanoparticles, including silver, gold, copper, titanium, zinc, and iron, exhibit antibacterial, antioxidant, and cytotoxic effects, making them valuable for various biomedical applications. The phytochemicals found in plant extracts, such as proteins, flavonoids, polyphenols, alkaloids, saponins, phenols, essential oils, and polyphenols, play a pivotal role in reducing metal ions biochemically, transforming them into metal nanoparticles, and concurrently providing a stabilizing cap for these synthesized nanoparticles [16].

In the present study, the color of the zinc oxide, chamomile, and green tea combination leaf extract solution mixture changed from yellowish to brownish-black during the green synthesis of zinc oxide nanoparticles. This alteration in color showed that zinc oxide nanoparticles had replaced the metallic zinc (Zn+) ions. Physical characterizations were then conducted on the obtained zinc oxide nanoparticles.

The BSA assay and the EA assay showed that zinc oxide nanoparticles have anti-inflammatory properties. The DPPH assay and the H₂O₂ assay used a change in color in the reaction mixture to show the antioxidant activity. As concentration increased, the inhibition percentage increased in a dose-dependent manner, and all results validated that the chamomile tea and green tea combination-mediated zinc oxide nanoparticles could be potentially used for delivering anti-inflammatory and antioxidant properties, respectively.

Nanoscale drug delivery of herbal medicines holds great promise for enhancing biological activity and addressing the limitations associated with chemical/synthetic drugs. Consequently, incorporating herbal remedies into nanodrug delivery systems has the potential to expand the utilization of herbal treatments and improve the management of various diseases. Herbal nanoparticles can find applications in preventive oral healthcare, dental prostheses, and teeth implantation, contributing to the prevention and treatment of oral diseases, including oral cancer, and promoting overall oral well-being. Additionally, extensive research is required to explore diverse combinations of herbal drugs and subsequently synthesize their nanoparticles. The innovations in herbal nanomedicine should undergo a systematic evaluation to assess their efficacy and suitability for patient use in the field of dentistry.

Limitations

Only the anti-inflammatory and antioxidant activity was assessed in vitro, which may not fully represent the potential of these green-synthesized nanoparticles to be used in varied fields of dentistry. Future studies to assess the cytotoxic potential of the nanoparticles are needed in order to utilize them to their full potential. In addition, this green-synthesized nanoparticle production should be translated into commercial production to minimize the side effects of metal nanoparticles produced otherwise. Additional research, encompassing in vivo experiments and molecular investigations, is imperative to corroborate the discovered results. Clinical trials are indispensable for evaluating the efficacy and safety of this combination within a clinical environment. Addressing these limitations in future research endeavors has the potential to advance our comprehension and utilization of these combinations in dentistry. As optimal concentration for maximum therapeutic effects varies from one herbal formulation to another, a specific concentration cannot be determined solely based on the present evaluations and requires further research.

## Conclusions

Within the limits of the present study, it can be concluded that the anti-inflammatory and antioxidant properties of chamomile and green tea combination-mediated zinc oxide nanoparticle formulation were higher than the controls in all concentrations, albeit better properties being exhibited at lower concentrations when compared with the controls; therefore, these nanoparticles synthesized using green methods could serve as a valuable supplement to current dental therapies and can be further evaluated for therapeutic application in oral lesions. Moreover, the integration of chamomile tea and green tea extracts, when employed in conjunction with other medications, offers the possibility of developing topical or systemic treatments for periodontal diseases. These formulations could equip clinicians with additional resources for effectively managing a range of periodontal conditions. In order to improve their properties, these green synthesized nanoparticles can also be added to dental materials or used to coat suture materials. They can also be used to create novel drugs that are more potent while being less toxic.
